# Identification and expression analysis of the *BnE2F/DP* gene family in *Brassica napus*

**DOI:** 10.3389/fpls.2025.1641897

**Published:** 2025-08-20

**Authors:** Min Shen, Lijuan Wei, Chun Fu

**Affiliations:** ^1^ Department of Agriculture, Forestry and Environmental Engineering, Changzhi Vocational and Technical College, Changzhi, Shanxi, China; ^2^ College of Agriculture and Biotechnology, Southwest University, Chongqing, China; ^3^ College of Life Sciences, Leshan Normal University, Leshan, Sichuan, China

**Keywords:** *Brassica napus*, E2F/DP, gene family, expression analysis, bioinformatics

## Abstract

*E2F/DP* (Eukaryotic Transcription Factor 2/Dimerization Partner) refers to a class of protein complexes that play a pivotal role in the regulation of gene transcription in eukaryotes. In higher plants, *E2F/DP* transcription factors are of vital significance in mediating responses to environmental stresses. Based on differences in their conserved structural domains, they can be categorized into three subgroups: E2F, DP, and DEL (DP-E2F-like). Studies on *E2F/DP* in plants are relatively scarce, and no research on *E2F/DP* in *Brassica* napus has been reported to date. Utilize bioinformatics approaches to identify the *BnE2F/DP* gene family in *Brassica napus*. Construct a phylogenetic tree for evolutionary relationship analysis, and analyze the chromosomal distribution, gene structure composition, types of cis-acting elements, as well as intragenomic and interspecific collinearity. Integrate transcriptome data with real-time quantitative PCR (RT-qPCR) technology to explore the expression patterns of *BnE2F/DP* gene family members across various tissues. Total 29 *BnE2F/DP* genes were identified in *Brassica napus* and classified into 8 sub-families. These genes were unevenly distributed across 15 chromosomes, with enrichment on chromosomes 3 and 13. Intragenomic collinearity analysis detected 35 pairs of collinear *BnE2F/DP* gene pairs. Interspecific analysis revealed that there were 9 pairs of orthologous gene pairs between *Brassica napus* and *Arabidopsis thaliana*. Cis-acting element analysis showed that members of the *BnE2F/DP* family harbored four types of cis-acting elements, which might be involved in multiple regulatory processes, including hormonal regulation, abiotic stress responses, light reactions, and growth and development. Transcriptome and quantitative PCR data analyses indicate that members of the *E2FC* subfamily are likely to actively regulate seed and embryo development and positively respond to various abiotic stresses.. The conserved motifs and gene structures within each *BnE2F/DP* subfamily are largely consistent, which indicates that *BnE2F/DP* plays a crucial role in the growth and development of rapeseed seeds and embryos, as well as in the response to various abiotic stresses.

## Introduction

*E2F/DP* (Eukaryotic Transcription Factor 2/Dimerization Partner) represents a class of protein complexes that play a pivotal role in the transcriptional regulation of genes in eukaryotes. It plays a crucial role for environmental stress responses (Mariconti et al., 2002). In eukaryotic cells, the RB/E2F signaling network orchestrates cell cycle progression, mediates mitotic division and DNA damage response, modulates cell size homeostasis, and ensures genomic integrity through dynamic phosphorylation by cyclin-dependent kinases (CDKs). Furthermore, this regulatory network mediates transcriptional reprogramming through sequence-specific interactions with cis-regulatory elements in target gene promoters. In planta, it plays a pivotal role in integrating photomorphogenic signaling with adaptive responses to abiotic/biotic stressors, thereby regulating spatiotemporal growth patterns, developmental transitions, and stress acclimation (Sozzani et al., 2006). In higher plants, *E2F/DP* transcription factors can be divided into three categories, namely E2F, DP, and DEL (DP - E2F - like), based on their conserved domains. The *E2F* gene group encompasses four functional domains: the retinoblastoma protein (RBR)-binding domain, the DNA-binding domain, the “mark box” domain, and the leucine-zipper dimerization domain. Typical E2Fs are able to act as either activators or repressors of gene expression. In contrast, non-typical E2Fs downregulate the expression of E2F-dependent genes by competing with typical E2Fs for DNA-binding sites. Notwithstanding recent advancements, current understanding of the E2Fs gene family evolutionary history, phylogenetic architectures, functional divergence among family members in plant species, and their roles within complex regulatory mechanism remains unclear. Currently, The E2F/DP transcription factor family in *Arabidopsis thaliana* represents the most comprehensively characterized member of this gene family among plant species. The *Arabidopsis thaliana E2F/DP* family consists of 8 members, including 3 types of canonical E2Fs (AtE2FA, AtE2FB, AtE2FC), 3 types of non-typical E2Fs (AtE2FD/DEL2, AtE2FE/DEL1, AtE2FF/DEL3), and 2 types of DP (Ramirez-Parra et al., 2004; Desvoyes et al., 2006). Functional studies have shown that E2Fs exhibit specific cooperative or antagonistic effects. The cross-regulation among various E2Fs has become part of the replication regulatory network in both animals and plants. In meristematic cells, AtE2F can activate cell-cycle genes to promote mitosis for cell proliferation. Cell proliferation in *Arabidopsis thaliana* depends on the transcriptional activation of *E2F/DP*. The deletion of the 3 typical *E2F/DP*s leads to plant sterility but does not affect vegetative growth (Magyar et al., 2005). The *DcE2F1* factor in carrot is one of the earliest *E2F* factors to be studied. *DcE2F1* is likely a homolog of *AtE2FA* in *Arabidopsis thaliana*. The phenotypes observed in *Arabidopsis thaliana* transformants with ectopic expression of DcE2F1 are highly similar to those of overexpressing AtE2FA. Conserved promotion of cell proliferation in embryonic and seedling tissues, coupled with ectopic hypocotyl formation, indicate its important role in the control of cell proliferation. In *Arabidopsis thaliana*, the cotyledon size of plants overexpressing AtE2FA or AtE2FB increases significantly, and the number of cells more than doubles. AtE2Fa acts downstream of the ERECTA kinase and is involved in regulating cell size and stomatal density. Studies on mammals also confirm that the E2F signaling pathway is crucial for cell growth and proliferation, demonstrating that *E2F/DP* plays a decisive role in the growth and development of organisms (Polager and Ginsberg, 2008). The *E2F/DP* gene family is extensively involved in plant growth, development, and responses to stress. Currently, *E2F/DP* genes have only been identified in a few plants such as *Arabidopsis thaliana*, moso bamboo, wheat, carrot, and tomato. There is no reported information on *Brassica napus* yet. For instance, salicylic acid is a key regulator in plant defense responses in *Arabidopsis thaliana*, the non-typical AtE2FE/DEL1 factor participates in the plant immune response and is found to inhibit the accumulation of salicylic acid to balance growth and defense. The promoters of *PheE2F/DP* transcription factors in moso bamboo harbor diverse cis-acting elements (Divya et al., 2024), which participate in leaf and root development, cell cycle regulation, and abiotic stresses responses. The expression of most *PheE2F/DP* genes is up-regulated under drought and salt stress (Li et al., 2021); The *E2F/DP* transcription factors in carrot regulate cell proliferation, size, and stomatal development, and are also involved in the growth and development processes of embryos and plants (Perrotta et al., 2021). Under drought stress, *E2F/DP* genes in wheat regulate the expression of downstream genes, maintain the balance of cell osmotic pressure, and enhance drought tolerance (Yu et al., 2022). Under drought stress, *Sorbus pohuashanensis E2F/DP* genes enhance the perception and response to water deficit by regulating the abscisic acid (ABA) signaling pathway, and regulate growth to adapt to a high-salt environment. Under drought stress, *Medicago sativa E2F/DP* genes maintain cell osmotic balance through multiple regulatory mechanisms, participate in hormone signal transduction pathways, and enhance stress tolerance, and the expression of four genes such as E2Fa is up-regulated by more than 2-fold under salt stress (Ma et al., 2014). In maize, the expression level of the *ZmE2F6* gene in the stem is significantly up-regulated after 3 hours of drought treatment and down-regulated at 6, 12, and 24 hours of treatment, indicating that ZmE2F6 responds to drought stress.

*Brassica napus*, as an important crop with great economic value, is widely used in fields such as food production and oil processing. However, the growth of *Brassica napus* relatively sensitive to water deficit, and drought stress has become a key factor severely constraining the increase in its yield (Wei et al., 2022). Therefore, identifying and harnessing stress-resistant genes in rapeseed through molecular breeding techniques holds great significance for the development of the rapeseed industry. In this study, 29 *BnE2F/DP* genes were comprehensively identified in the *Brassica napus* genome. Using bioinformatics and other methods, we performed an in-depth analysis of the gene structures, physicochemical properties, chromosome locations, conserved motifs, cis-acting elements, intragenomic collinearity, and phylogenetic evolutionary characteristics of the *BnE2F/DP* gene family members in rapeseed. By integrating transcriptome sequencing and real-time fluorescence quantitative PCR (RT-qPCR) techniques, we analyzed the expression patterns of the *BnE2F/DP* gene family in different tissues of *Brassica napus*. The findings of this study not only enhance our knowledge of the biological functions of the *BnE2F/DP* gene family in *Brassica napus*, but also provide important gene resources and a theoretical basis for stress-resistant molecular breeding of rapeseed. Moreover, they are anticipated to expedite the process of molecular-assisted selection for stress-resistant in rapeseed breeding.

## Materials and methods

### Materials

The material used in this experiment is *Brassica napus* “Zhongshuang 11”, which was provided by the College of Agronomy and Biotechnology, Southwest University. The seeds of *Brassica napus* were sown in the field. After the rapeseed entered the flowering stage, young seeds 10 to 49 days post-flowering were collected. They were rapidly frozen in liquid nitrogen and then used for the qRT-PCR experiment. Three replicates were set for each treatment.

### Methods

#### Identification of *BnE2F/DP* genes in the *Brassica napus* genome

The genomic sequences and *E2F/DP* protein sequences of *Arabidopsis thaliana* were downloaded from the TAIR database (https://www.arabidopsis.org/), and the genomic sequences, protein sequences, and gene annotation information of *Brassica napus* were downloaded from the BRAD (http://www.brassicadb.cn/) database (Berardini et al., 2015; Li et al., 2024). Firstly, a two-way BLAST comparison was performed between the protein sequences of *Arabidopsis thaliana* and *Brassica napus* to preliminarily screen out the candidate genes of the *BnE2F/DP* gene family in *Brassica napus* (Jia et al., 2021). Thereafter, to ensure the accuracy and reliability of the screening results, we applied a dual-screening approach using BLAST and HMMER, and finally determined the members of the *BnE2F/DP* gene family.

#### Analysis of chromosomal localization and physicochemical properties of *BnE2F/DP* proteins in *Brassica napus*

The distribution map of the *BnE2F/DP* genes of *Brassica napus* in the genome was generated using the software TBtools. We carried out prediction analysis of indicators including the amino acid length, theoretical isoelectric point, relative molecular mass, average hydrophilicity, instability coefficient, and aliphatic index of the candidate proteins of the *BnE2F/DP* genes in *Brassica napus* using the online software ExPASy (Li et al., 2022). The subcellular localization analysis of the *BnE2F/DP* proteins in *Brassica napus* was predicted using the online software CELLO v2.5 (Li et al., 2009).

#### Phylogenetic analysis of members of *BnE2F/DP* gene family

To gain an in-depth understanding of the evolutionary relationships among the members of the *BnE2F/DP* gene family, we used the Muscle software, a bioinformatics tool, to conduct a multiple sequence alignment analysis of the *BnE2F/DP* proteins in *Arabidopsis thaliana* and *Brassica napus* (Cao et al., 2024). Subsequently, the iqtree software was employed to screen for the optimal tree-building model. Then, the raxml software was utilized to construct a phylogenetic tree (Safder et al., 2022). Finally, the visualization of the phylogenetic tree was achieved using the R software package.

#### Analysis of gene structures and conserved motifs of *BnE2F/DP* gene family

For the analysis of gene structures, the online software Gene Structure Display Server was employed to analyze the exon/intron distribution of the *BnE2F/DP* genes in *Brassica napus* and draw gene structure diagrams (Pu et al., 2020). We employed the Clustal X software to perform sequence alignment on the finally determined *BnE2F/DP* protein sequences in *Brassica napus*. Subsequently, the Jalview software was used to analyze and present the sequence features of the members of the *BnE2F/DP* family in *Brassica napus* visually (Li et al., 2014). Meanwhile, the MEME online website was utilized to analyze the conserved motifs of the *BnE2F/DP* proteins (Bailey et al., 2009), providing a basis for in-depth exploration of the structures and functions of this gene family.

#### Analysis of cis-acting elements in the promoters of *BnE2F/DP* gene family

We used the bedtools and seqkit software to extract the promoter region sequences of 1500 bp upstream of the start codon of the members of the *BnE2F/DP* gene family in *Brassica napus*. The cis-acting elements in this promoter region were predicted through the PlantCARE5 online website (Liu et al., 2014; Biłas et al., 2016). Finally, the R software package was utilized to visually display the prediction results.

#### Identification of duplication events and collinearity analysis of *BnE2F/DP* gene family

To identify the gene duplication events of the *BnE2F/DP* genes in *Brassica napus*, we used the MCScanX software to identify the types of gene duplication (Cannon et al., 2004). Meanwhile, the orthofinder software was employed to analyze the gene collinearity between *Arabidopsis thaliana* and *Brassica napus*, and the data were output with the default parameters (Tamura et al., 2021). Finally, the circos software was utilized to visually display the gene duplication events and collinearity results for an intuitive presentation of the relevant characteristics (International Brachypodium Initiative, 2010; Hu et al., 2015).

#### Analysis of the expression patterns of the *BnE2F/DP* gene family

Based on the publicly available transcriptome data of *Brassica napus*, the FPKM (Fragments Per Kilobase of transcript per Million mapped reads) value analysis method was employed to investigate the expression status of genes in the *BnE2F/DP* family of *Brassica napus* in different tissues and organs. Subsequently, we used the Heatmap program in the TBtools software to draw a heatmap of the expression patterns of the members of the *BnE2F/DP* gene family in different tissues and organs of *Brassica napus*, aiming to intuitively present the gene expression characteristics (Gao et al., 2014; Wai et al., 2022).

#### qRT-PCR analysis of *BnE2F/DP* gene family

To identify the expression of the *BnE2F/DP* genes in different tissues of *Brassica napus*, RNA was extracted and then reverse-transcribed into cDNA after materials collection. Eight *BnE2F/DP* genes that were representative in seed expression were screened out from the heatmap. Real-time fluorescence quantitative PCR primers for the members of the *BnE2F/DP* gene family were designed using the Primer-BLAST tool available on NCBI (Berardini et al., 2015) (**Table 1**). The expression levels of these eight *BnE2F/DP* genes in the seeds at the above five stages were determined by real-time PCR. Bn-actin was used as an internal reference gene to calculate the expression levels of the target genes (Filichkin et al., 2010; Sharma et al., 2017).

**Table 1:** Primer sequences of members of the *BnE2F/DP* family.

Gene name	Forward primer	Reverse primer
BnDPA-2	GTGTGCAAAAGAAGGCTGTT	ACGGATGCTTACAAACTCTAACAAG
BnDPB-3	CTCGTCCAGACTCGTCCTCA	GCTGTTGTTATTGTGACCGTTGT
BnE2FC-2	CACCCACTGATCCGCTTCAA	ATTTGCGGCGATTGTGCATT
BnE2FC-1	ATCCGGGGAAGATCCGAGTC	AATTTGCGGCGATGGAAGCG
BnE2FA-6	GATGTGGTTGCTGCTCCATCT	TGAGGAACTGACTGGTTTCCTT
BnDPA-4	TTCGCCTTCCTCTACACATCAA	AGCGATTATCATTGGTTCCCGA
BnDPB-4	CTCACAGTTTCGTGCCGGA	GAGCATTTACTGGTTGCTGAGGC
BnDPB-1	CAACAACCGCCAACATGACG	AAAGGCACTGTCGGTAGCAG
Actin	TGGGTTTGCTGGTGACGAT	TGCCTAGGACGACCAACAATACT

#### Expression analysis and qRT-PCR validation of *BnE2F/DP* gene family under abiotic stress in *Brassica napus*

The transcriptome sequencing data of *Brassica napus* under salt stress, ABA stress, and drought stress were downloaded from the NCBI database. The Fragments Per Kilobase of transcript per Million mapped reads(FPKM) expression data of *BnE2F/DP* genes were extracted, and clustering heatmaps were drawn using TBtools software.

The *Brassica napus* plants were cultivated in pots in a greenhouse under a 16h/8h photoperiod at 25°C. When the plants grew to the four-leaf and one-bud stage, those with uniform growth vigor were selected as experimental materials. Drought stress was simulated by watering the substrate with 20% (w/v) polyethylene glycol 6000 (PEG6000), while the control group was watered with clear water. Leaf tissues were collected at 0, 6, 12, 24, 48, and 72 hours after treatment. Salt stress was simulated by watering the plant substrate with 200 mmol/L NaCl, while the control group was watered with clear water. After treatment, leaf tissues were collected at 0, 1, 3, 6, 12, and 24 hours. Leaves of the plants were sprayed with 100 μmol/L ABA (abscisic acid), and leaf samples were collected at 0, 1, 3, 6, 12, and 24 hours after treatment. All samples were stored in liquid nitrogen after collection, and stored for subsequent use. Each treatment was repeated three times.

## Results

### Identification and physicochemical property analysis of the *BnE2F/DP* gene family in *Brassica napus*

By screening the *Brassica napus* database, a total of 29 members of the *BnE2F/DP* gene family were identified. Then, based on the phylogenetic relationships between these 29 *BnE2F/DP* genes in *Brassica napus* and those in *Arabidopsis thaliana*, as depicted in the phylogenetic tree, they were designated as *BnE2FA-1* to *BnDPA-4* in a clockwise sequence within the same subfamily.

**Figure 1:**
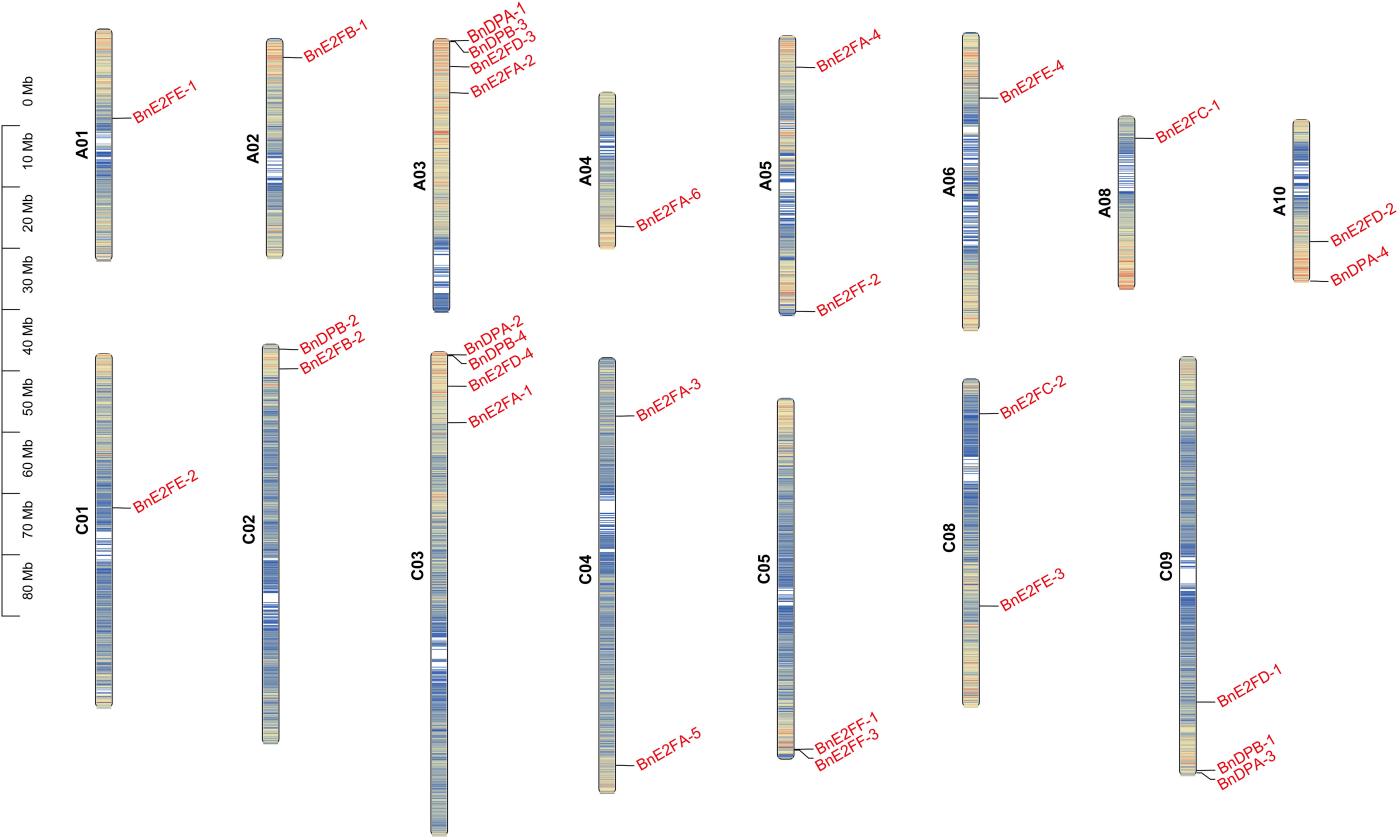
The distribution of *BnE2F/DP* gene on 15 chromosomes in *Brassica napus*.

A visual analysis of the 29 *BnE2F/DP* family members was conducted based on chromosomal location information (**Figure 1**). The results revealed that these genes were unevenly distributed across the chromosomal scaffolds, which play an important role in organizing the spatial arrangement of genes. Their presence was detected on 15 chromosomes, excluding chromosomes 7, 9, 16, and 17. Both chromosome 3 and chromosome 13 harbored four genes each, and most of the genes were located at the terminal regions of the chromosomes.

The analysis of physicochemical properties (**Table 2**) showed that the theoretical isoelectric points (pI) of the *BnE2F/DP* genes ranged from 4.67 (BnE2FD-4) to 9.8 (BnDPA-2). Moreover, the relative molecular masses of these genes exhibited a wide range, being between 11860.58 Da (BnE2FF-3) and 107944.84 Da (BnDPB-1). Additionally, the lengths of amino acids varied from 290 amino acids (aa) (BnDPA-4) to 970 aa (BnDPB-1). Subcellular localization indicated that only the BnE2FF-3 protein was located in the cytoplasm, while the rest were all in the nucleus. Furthermore, the analysis revealed that the instability coefficients of all the proteins were greater than 40, classifying them as unstable proteins. The aliphatic amino acid index ranged from 61.15 to 103.98, and the grand average of hydropathicity (GRAVY) values were between -0.886 and -0.289. According to the criteria that proteins with a GRAVY value below 0 are considered hydrophilic, these proteins were determined to be hydrophilic proteins.

**Table 2:** Basic information of *BnE2F/DP* gene.

Gene name	Sequence ID	Chronmosome position	Amino Acids (aa)	Molecular Weight (Da)	Theoretical Isoelectric point	Sub-cellular localization	Instability index	Aliphatic index	Grand Average of hydropathicity
*BnE2FE-1*	BnaA01T0224200ZS	A01	389	43807.96	7.26	nucl	53.07	70.23	-0.6
*BnE2FB-1*	BnaA02T0056600ZS	A02	349	39595.19	9.07	nucl	48.28	81.86	-0.563
*BnDPA-1*	BnaA03T0006700ZS	A03	283	32345.8	7.72	nucl	56.79	81.27	-0.68
*BnDPB-3*	BnaA03T0010200ZS	A03	361	39922.18	5.67	nucl	59.73	66.95	-0.81
*BnE2FD-3*	BnaA03T0094100ZS	A03	517	57601.15	5.04	nucl	51.21	69.57	-0.71
*BnE2FA-2*	BnaA03T0172900ZS	A03	464	50881.61	4.83	nucl	61.96	76.47	-0.57
*BnE2FA-6*	BnaA04T0231600ZS	A04	487	52952.83	4.91	nucl	55.91	74.48	-0.61
*BnE2FA-4*	BnaA05T0089000ZS	A05	481	52256.17	4.79	nucl	53.2	77.42	-0.54
*BnE2FF-2*	BnaA05T0495400ZS	A05	342	38621.51	6.77	nucl	57.2	79.82	-0.62
*BnE2FE-4*	BnaA06T0169600ZS	A06	384	43397.44	7.19	nucl	51.66	72.42	-0.73
*BnE2FC-1*	BnaA08T0042700ZS	A08	365	41318.49	5.63	nucl	55.9	76.63	-0.69
*BnE2FD-2*	BnaA10T0166000ZS	A10	443	49045.48	4.75	nucl	54.82	73.3	-0.73
*BnDPA-4*	BnaA10T0296600ZS	A10	290	32886.34	7.7	nucl	54.42	75.59	-0.67
*BnE2FE-2*	BnaC01T0287900ZS	C01	561	63043.05	6.25	nucl	48.5	81.27	-0.51
*BnDPB-2*	BnaC02T0010200ZS	C02	338	37378.39	7.24	nucl	46.64	61.15	-0.89
*BnE2FB-2*	BnaC02T0066000ZS	C02	349	39551.05	9.12	nucl	47.73	80.74	-0.59
*BnDPA-2*	BnaC03T0010100ZS	C03	200	23482.62	9.8	nucl	43.66	92.05	-0.60
*BnDPB-4*	BnaC03T0014300ZS	C03	320	35603.57	6.33	nucl	55.58	66.69	-0.84
*BnE2FD-4*	BnaC03T0106500ZS	C03	459	50949.47	4.67	nucl	53.34	70.7	-0.76
*BnE2FA-1*	BnaC03T0201300ZS	C03	469	51248.96	4.69	nucl	61.5	77.76	-0.54
*BnE2FA-3*	BnaC04T0105100ZS	C04	482	52511.61	4.93	nucl	55.22	78.28	-0.55
*BnE2FA-5*	BnaC04T0546900ZS	C04	487	53004.01	4.98	nucl	57.66	75.69	-0.59
*BnE2FF-1*	BnaC05T0558400ZS	C05	302	33938.96	6.61	nucl	52.32	69.4	-0.71
*BnE2FF-3*	BnaC05T0561200ZS	C05	103	11860.58	6.83	Cyto/nucl	60.63	103.98	-0.29
*BnE2FC-2*	BnaC08T0055000ZS	C08	377	42512.76	5.63	nucl	62.55	76.53	-0.68
*BnE2FE-3*	BnaC08T0285500ZS	C08	385	43439.54	7.18	nucl	49.4	73.51	-0.70
*BnE2FD-1*	BnaC09T0449800ZS	C09	443	49032.54	4.77	nucl	55.1	73.3	-0.73
*BnDPB-1*	BnaC09T0610700ZS	C09	970	107944.84	4.94	nucl	51.74	69.04	-0.74
*BnDPA-3*	BnaC09T0617200ZS	C09	318	36062.09	8.12	nucl	55.66	79.03	-0.50

### Phylogenetic analysis of the *BnE2F/DP* family in *Brassica napus*

A phylogenetic tree was constructed using 29 *BnE2F/DP* protein sequences from *Brassica napus* and 8 At*E2F/DP* protein sequences from *Arabidopsis thaliana*. Referring to the grouping rules adopted by Cubas in the research on *Arabidopsis thaliana*, which were mainly based on the sequence similarity and structural characteristics of the proteins, the *BnE2F/DP* members in *Brassica napus* were classified into eight sub-families, namely *DPA, DPB, E2FA, E2FB, E2FC, E2FD, E2FE*, and *E2FF*.

**Figure 2:**
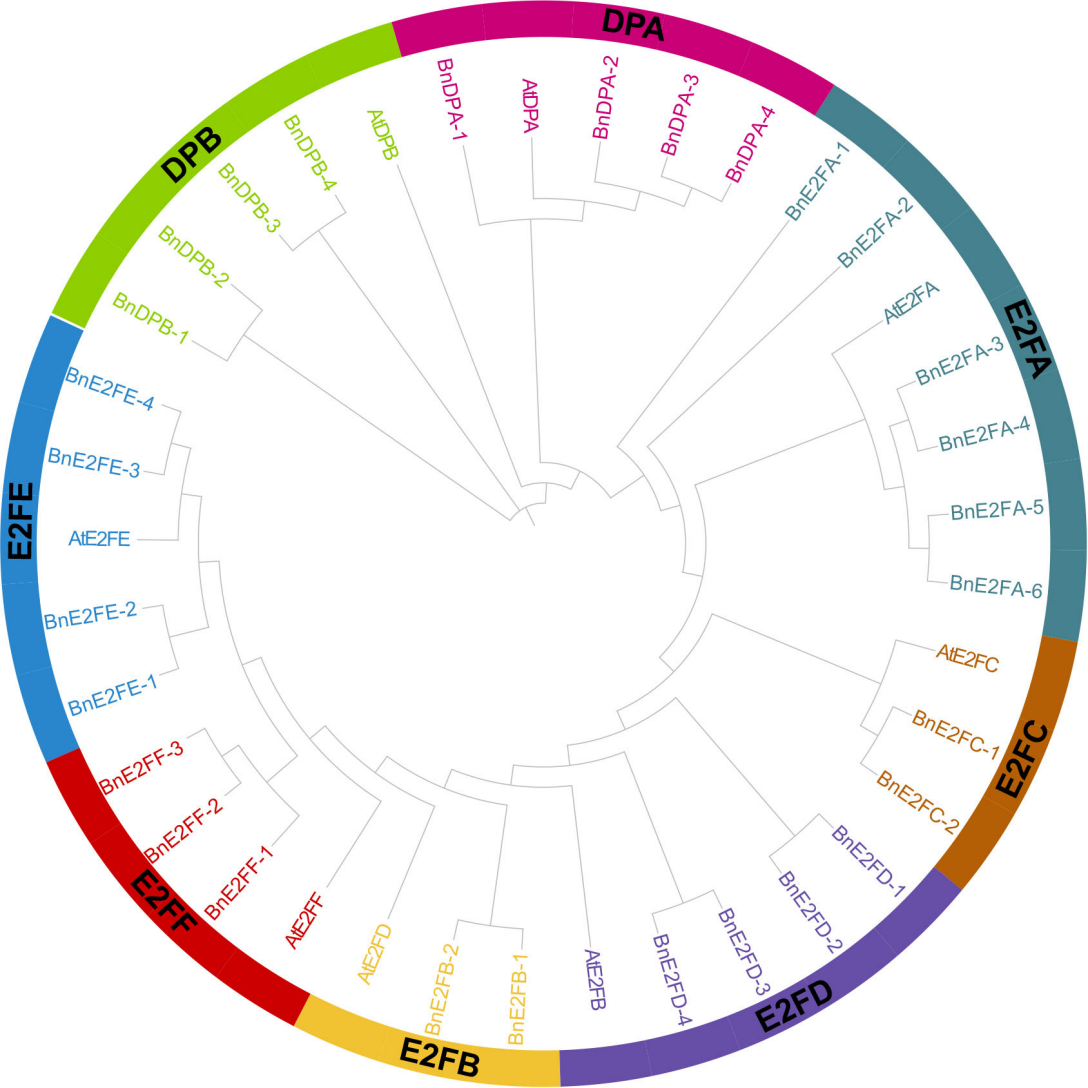
Analysis of the phylogenetic relationship and gene structure of BnE2F/DP genes.

The results of phylogenetic tree analysis demonstrated that among the eight sub-families, the E2FA sub-family had the largest number of members, totaling seven in number. In detail, six members were from *Brassica napus* and one from *Arabidopsis thaliana*. Conversely, the E2FB and E2FC sub-families had the smallest number of members, each having only three members, comprising two from *Brassica napus* and one from *Arabidopsis thaliana*. It is postulated that the gene members of E2FA fulfill more pivotal functions in plant growth, development, and stress-tolerance responses. Furthermore, the expansion of the *BnE2F/DP* gene family might be attributable to the increase of gene copy numbers within this sub-family. Moreover, each clade contained *E2F/DP* protein members from both *Brassica napus* and *Arabidopsis thaliana*. This finding implies that there exists a definite phylogenetic affinity and a shared ancestry between the two species in the evolution of *E2F/DP* genes, suggesting a remarkable degree of continuity and conservation of this transcription factor family throughout the evolutionary course (**Figure 2**).

### Analysis of gene structure and conserved motifs of *BnE2F/DP* genes in *Brassica napus*

**Figure 3:**
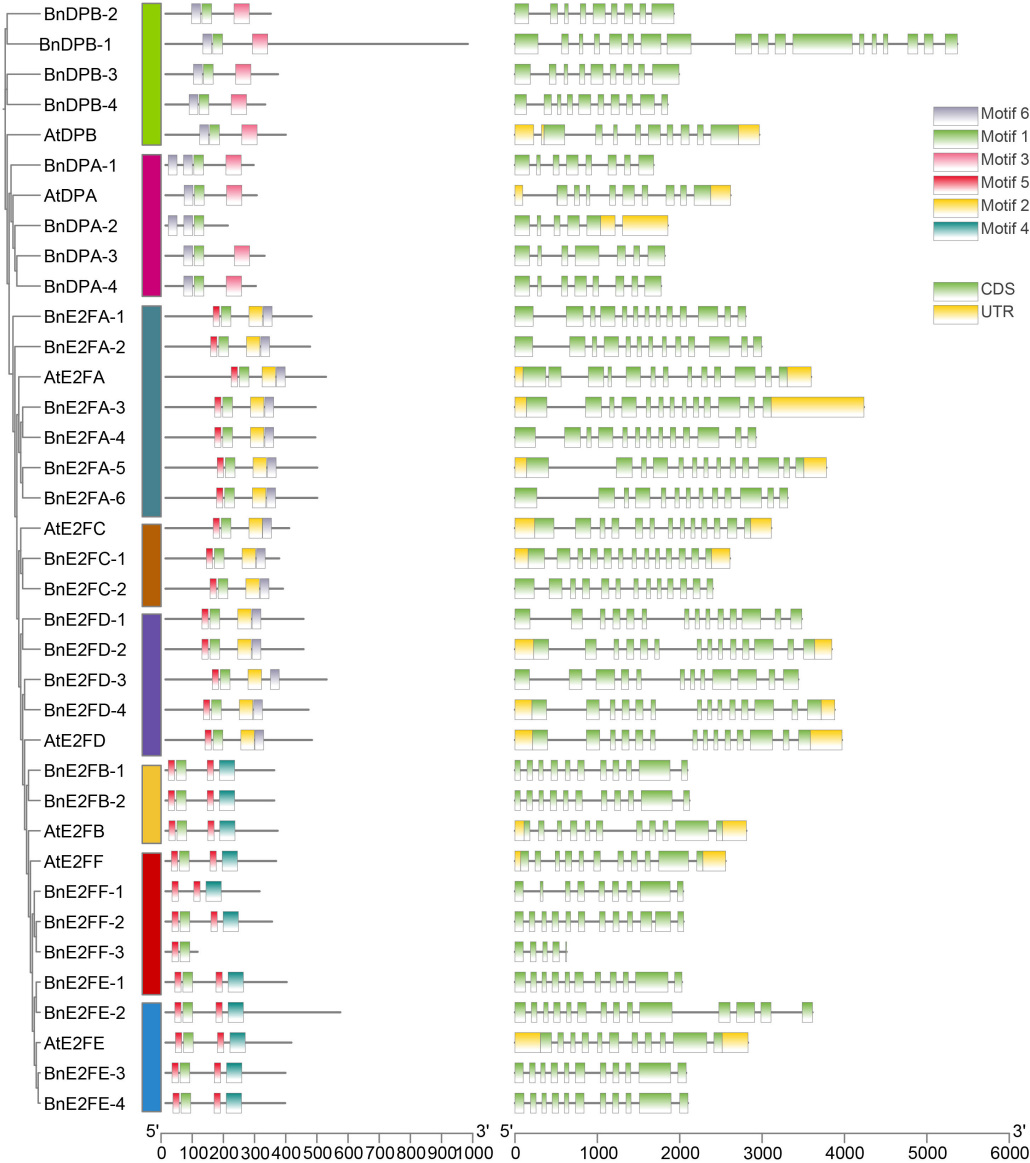
Analysis of the phylogenetic relationship and gene structure of *BnE2F/DP* genes.

The gene structures of *BnE2F/DP* in *Brassica napus* and the conserved motifs of their corresponding proteins were separately analyzed. As presented in **Figure 3**, the gene structures within the *BnE2F/DP* family are intricate, with the exon count ranging from 5 to 18. Members featuring similar structures mainly aggregate within the same clade. Specifically, for instance, members of both the BnE2FA and BnE2FC families consistently possess 13 exons. The number of exons in the BnDPB sub-family ranges from 9 and 18. In the BnDPA subfamily, the exon numbers are relatively smaller. Specifically, BnDPA-1 and BnDPA-4 contain 8 exons, while BnDPA-2 has 5 exons, and BnDPA-3 has 9.

Conserved motifs are highly similar sequences retained during long-term evolution. Analysis of the proteins of 29 *BnE2F/DP* family members in *Brassica napus* revealed that all contain the E2F/TDP-2 conserved domain, indicating this motif is a highly conserved and critical region within the family, preserved throughout evolution. Using MEME software, we predicted conserved regions in the amino acid sequences of the *BnE2F/DP* members (**Figure 3**), identifying six distinct motifs. Motif 1 was conserved across all family members, suggesting it corresponds to the E2F/TDP-2 domain. Notably, marked variations in motif composition were observed across subfamilies. The DPB subfamily contained Motifs 1, 3, and 6; the DPA subfamily contained Motifs 1 and 6; the E2FA, E2FC, and E2FD subfamilies contained Motifs 5, 1, 2, and 6; the E2FB and E2FE subfamilies contained Motifs 5, 1, and 4; and the E2FF subfamily contained Motifs 5 and 1. Collectively, these results indicate that the higher the sequence similarity of the *BnE2F/DP* genes, the more similar their structures. Members within the same subfamily shared conserved motif compositions and arrangement, supporting the hypothesis of conserved biological functions of the subfamily members (**Figure 3**).

### Analysis of cis-acting elements in the promoters of *BnE2F/DP* gene family in *Brassica napus*

**Figure 4:**
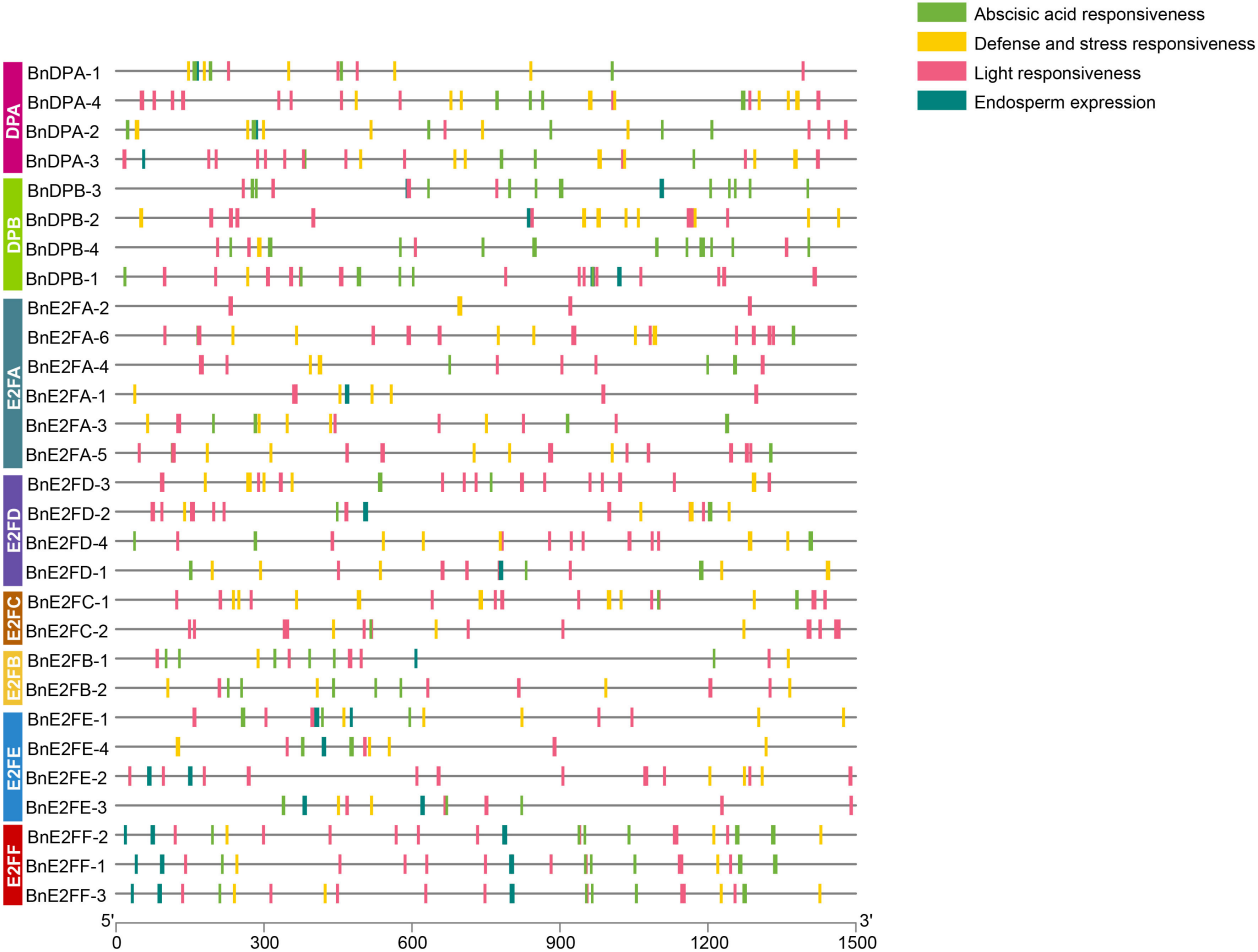
Analysis of cis-acting elements in the promoter region of *BnE2F/DP* genes.

Cis-acting elements bind to transcription factors to regulate gene expression efficiency. Identifying and analyzing the cis-acting elements in the promoter regions of 29 *BnE2F/DP* genes can aid in predicting their functions. Specially, the nucleotide sequences of the promoter regions, located 1500 bp upstream of the ATG start codon for each of the 29 *BnE2F/DP* genes, were retrieved from the NCBI database. The PlantCARE 5, a plant cis-acting element analysis tool, was used to predict the cis-acting elements in these sequences. Subsequently, elements with potential biological functions were identified. The positional distribution patterns of each element in the sequences are detailed in **Figure 4**. As shown in the figure, the promoters of all *BnE2F/DP* genes contain multiple cis-acting elements. Among these, light response cis-elements are the most abundant, with each of the 29 genes harboring between 3-13 such elements. Except for BnDPB-2, BnE2FA-2, BnE2FA-1, and BnE2FE-2, 25 *BnE2F/DP* genes possess hormone-responses cis-acting elements primarily including those responsive to abscisic acid, methyl jasmonate, gibberellin, salicylic acid and auxin. Except for BnDPB-3, 28 *BnE2F/DP* genes contain cis-acting elements associated with abiotic stresses, including those involved in anaerobic induction, low-temperature response, drought induction, and defense and wounding stimuli. Additionally, 18 *BnE2F/DP* genes harbor cis-acting elements related to growth and development, primarily including those associated with endosperm-specific expression, meristematic tissue expression, flavonoid biosynthesis regulation, cell cycle regulation, and root-specific regulatory responses.

### Analysis of collinearity and duplication events of the *BnE2F/DP* gene family in *Brassica napus*

Gene duplication events represent a critical mechanism for the rapid expansion of plant gene families. Given that *Brassica napus* has undergone whole-genome duplication during its evolutionary history, the presence of duplicated genes is likely. Using the MCScanX software, we analyzed duplication patterns in the *BnE2F/DP* gene family and found that only one gene underwent dispersed duplication, one remained as a single copy, and 27 originated from whole-genome duplication events. This strongly indicated that whole-genome duplication plays a dominant role in the expansion process of this gene family. Specifically, BnE2FF-2 resulted from dispersed duplication, whereas BnE2FF-3 remained a single-copy gene.

**Figure 5:**
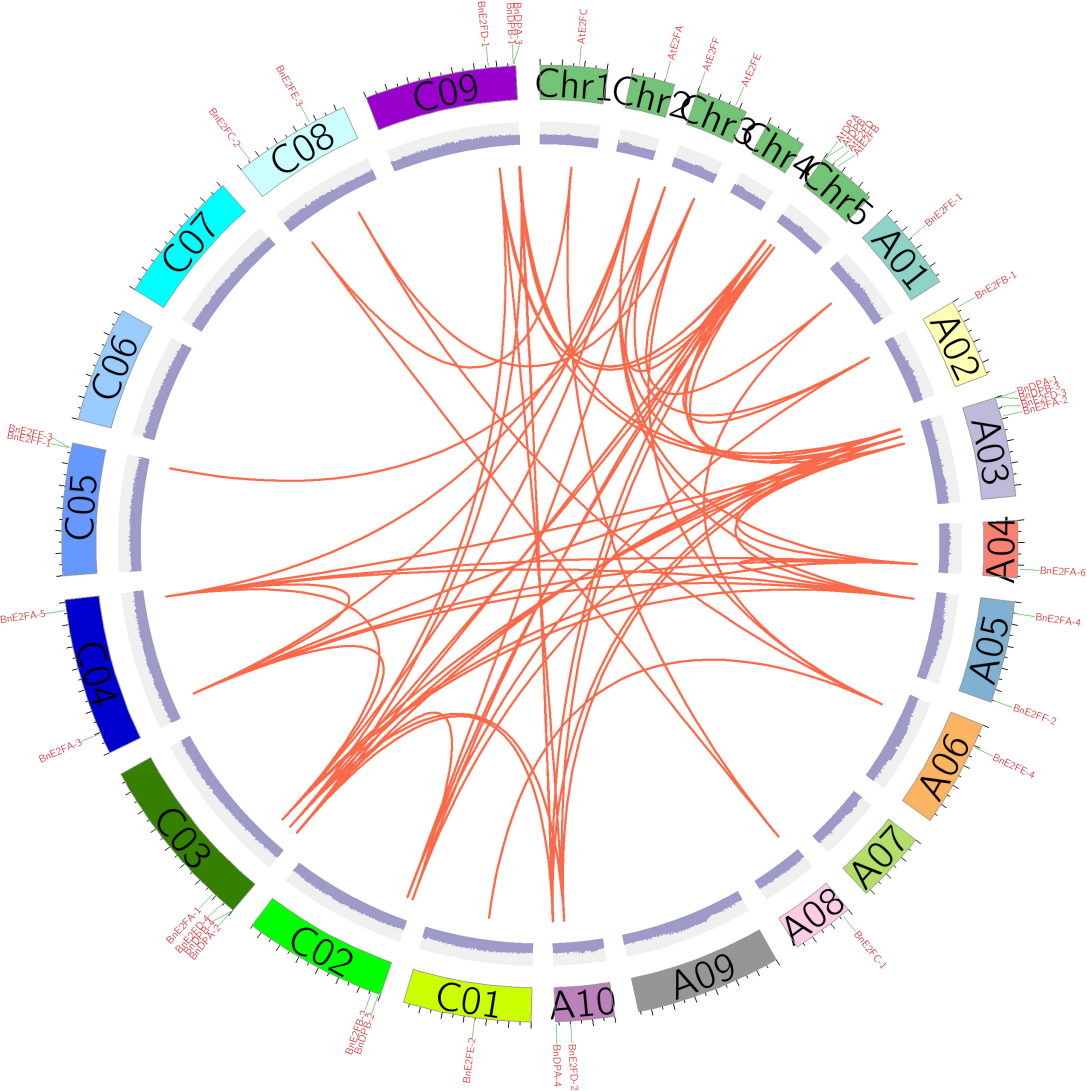
Correlation analysis of *BnE2F/DP* genes.

To further investigate the evolutionary relationships within the *BnE2F/DP* gene family, TBtool was used to visualize collinear regions of the *BnE2F/DP* genes in *Brassica napus* and analyze both intraspecific and interspecific collinearities. The results revealed one pair of collinear genes within the At*E2F/DP* family in *Arabidopsis thaliana*. In the *BnE2F/DP* gene family of *Brassica napus*, 35 pairs of intraspecific collinear gene were identified, which were distributed across 14 chromosomes. Except for BnE2FF-1, BnE2FF-3, and BnDPB-1, the remaining family members showed significant intraspecific collinearity. This result suggests that gene duplication events have occurred in the *E2F/DP* gene family, and it is hypothesized that the expansion of the number of family members may be accomplished through these duplication events. Additionally, examples exist where one gene is collinear with multiple genes, indicating that these genes play critical and irreplaceable roles in maintaining *Brassica napus* growth, development and physiological activities, with their functions being highly conserved. The prevalence of extensive collinear gene pairs within the family supports the hypothesis of functional redundancy. Gene duplication not only increase gene copy numbers but also enhance plant adaptability to environmental stresses (**Figure 5**).

To investigate the selection pressure acting on *BnE2F/DP* during evolution, we performed a Ka/Ks analysis on 35 intraspecific duplicated gene pairs and analyze syntenic blocks between *Arabidopsis thaliana* and *Brassica napus*. Specifically, Ka/Ks ratios > 1 indicate positive selection driving adaptive evolution; ratios =1 reflect neutral selection, where natural selection does not influence gene evolution; ratios < 1 suggest purifying selection maintaining functional conservation. The analysis results revealed that the Ka/Ks values of the 35 duplication events in *Brassica napus* ranged from 0.0456798 to 0.397235 (**Table 3**), the analysis identify nine orthologous genes pairs between the two species, with Ka/Ks ratios ranging from 0.159425 to 0.594844 (**Table 4**), all of which were less than 1. This demonstrates that the duplicated genes have undergone purifying selection during evolution and the orthologous *E2F/DP* genes have also undergone purifying selection during interspecific evolution.

**Table 3:** Analysis of Ka/Ks ratio of *BnE2F/DP* repeat genes in *Brassica napus*.

Gene pairs	Ka	Ks	Ka/Ks	Selective pressure	Duplicate type
BnE2FE-1-BnE2FE-4	0.0546034	0.421464	0.129557	Purifying selection	Interchromosomal
BnE2FB-1-BnE2FB-2	0.0112726	0.120479	0.0935646	Purifying selection	Interchromosomal
BnDPA-1-BnDPA-4	0.0596839	0.411673	0.144979	Purifying selection	Interchromosomal
BnDPA-1-BnDPA-2	0.0919465	0.231466	0.397235	Purifying selection	Interchromosomal
BnDPA-1-BnDPA-3	0.0589233	0.386755	0.152353	Purifying selection	Interchromosomal
BnDPB-3-BnDPB-2	0.142025	0.417739	0.339984	Purifying selection	Interchromosomal
BnDPB-3-BnDPB-4	0.0184681	0.0874147	0.21127	Purifying selection	Interchromosomal
BnE2FD-3-BnE2FD-2	0.0663793	0.253021	0.262347	Purifying selection	Interchromosomal
BnE2FD-3-BnE2FD-4	0.0316302	0.115395	0.274102	Purifying selection	Interchromosomal
BnE2FD-3-BnE2FD-1	0.0684454	0.244836	0.279556	Purifying selection	Interchromosomal
BnE2FA-2-BnE2FA-6	0.0848051	0.355889	0.238291	Purifying selection	Interchromosomal
BnE2FA-2-BnE2FA-4	0.0819038	0.27476	0.298092	Purifying selection	Interchromosomal
BnE2FA-2-BnE2FA-1	0.0295699	0.118022	0.250547	Purifying selection	Interchromosomal
BnE2FA-2-BnE2FA-3	0.0825192	0.316581	0.260657	Purifying selection	Interchromosomal
BnE2FA-2-BnE2FA-5	0.0903916	0.325096	0.278046	Purifying selection	Interchromosomal
BnE2FA-6-BnE2FA-4	0.079672	0.303437	0.262565	Purifying selection	Interchromosomal
BnE2FA-6-BnE2FA-1	0.0886543	0.337323	0.262817	Purifying selection	Interchromosomal
BnE2FA-6-BnE2FA-3	0.0831487	0.296878	0.280076	Purifying selection	Interchromosomal
BnE2FA-6-BnE2FA-5	0.0249927	0.0745803	0.335111	Purifying selection	Interchromosomal
BnE2FA-4-BnE2FA-1	0.0986496	0.320396	0.307899	Purifying selection	Interchromosomal
BnE2FA-4-BnE2FA-3	0.0250524	0.0693633	0.361176	Purifying selection	Interchromosomal
BnE2FA-4-BnE2FA-5	0.0858963	0.28882	0.297404	Purifying selection	Interchromosomal
BnE2FE-4-BnE2FE-2	0.0945137	0.510167	0.18526	Purifying selection	Interchromosomal
BnE2FE-4-BnE2FE-3	0.0114259	0.0893741	0.127844	Purifying selection	Interchromosomal
BnE2FC-1-BnE2FC-2	0.00831829	0.0467297	0.178009	Purifying selection	Interchromosomal
BnE2FD-2-BnE2FD-4	0.0673391	0.257757	0.26125	Purifying selection	Interchromosomal
BnE2FD-2-BnE2FD-1	0.0117697	0.0409083	0.28771	Purifying selection	Interchromosomal
BnDPA-4-BnDPA-2	0.150763	0.636255	0.236954	Purifying selection	Interchromosomal
BnDPA-4-BnDPA-3	0.00884364	0.193601	0.0456798	Purifying selection	Interchromosomal
BnDPB-2-BnDPB-4	0.138254	0.434873	0.317918	Purifying selection	Interchromosomal
BnDPA-2-BnDPA-3	0.152244	0.622893	0.244414	Purifying selection	Interchromosomal
BnE2FD-4-BnE2FD-1	0.0645329	0.25672	0.251375	Purifying selection	Interchromosomal
BnE2FA-1-BnE2FA-3	0.0939614	0.353875	0.265521	Purifying selection	Interchromosomal
BnE2FA-1-BnE2FA-5	0.0879968	0.31679	0.277776	Purifying selection	Interchromosomal
BnE2FA-3-BnE2FA-5	0.089422	0.282396	0.316655	Purifying selection	intrachromosomal

**Table 4:** Analysis of Ka/Ks ratio of homologous genes *BnE2F/DP* in *Brassica napus* and *AtE2F/DP* in *Arabidopsis thaliana*.

Gene pairs	Ka	Ks	Ka/Ks	Differentiation time	Differentiation time(My)
BnaA02T0056600ZS-AT5G14960	0.203816	0.391478	0.520632	13049266.67	13.04926667
BnaA05T0495400ZS-AT3G01330	0.2178	0.491804	0.442859	16393466.67	16.39346667
BnaA05T0495400ZS-AT3G48160	0.511142	2.42439	0.210833	80813000	80.813
BnaC02T0066000ZS-AT5G14960	0.200613	0.337253	0.594844	11241766.67	11.24176667
BnaC05T0558400ZS-AT3G01330	0.202917	0.398834	0.508775	13294466.67	13.29446667
BnaC05T0558400ZS-AT3G48160	0.568935	3.56866	0.159425	118955333.3	118.9553333
BnaC05T0561200ZS-AT3G01330	0.184482	0.550076	0.335376	18335866.67	18.33586667
BnaC05T0561200ZS-AT3G48160	0.379269	1.63207	0.232386	54402333.33	54.40233333
BnaC09T0610700ZS-AT5G02470	0.504838	NA	NA	NA	NA

### Analysis of the tissue expression patterns of members of the *BnE2F/DP* gene family in *Brassica napus*

**Figure 6:**
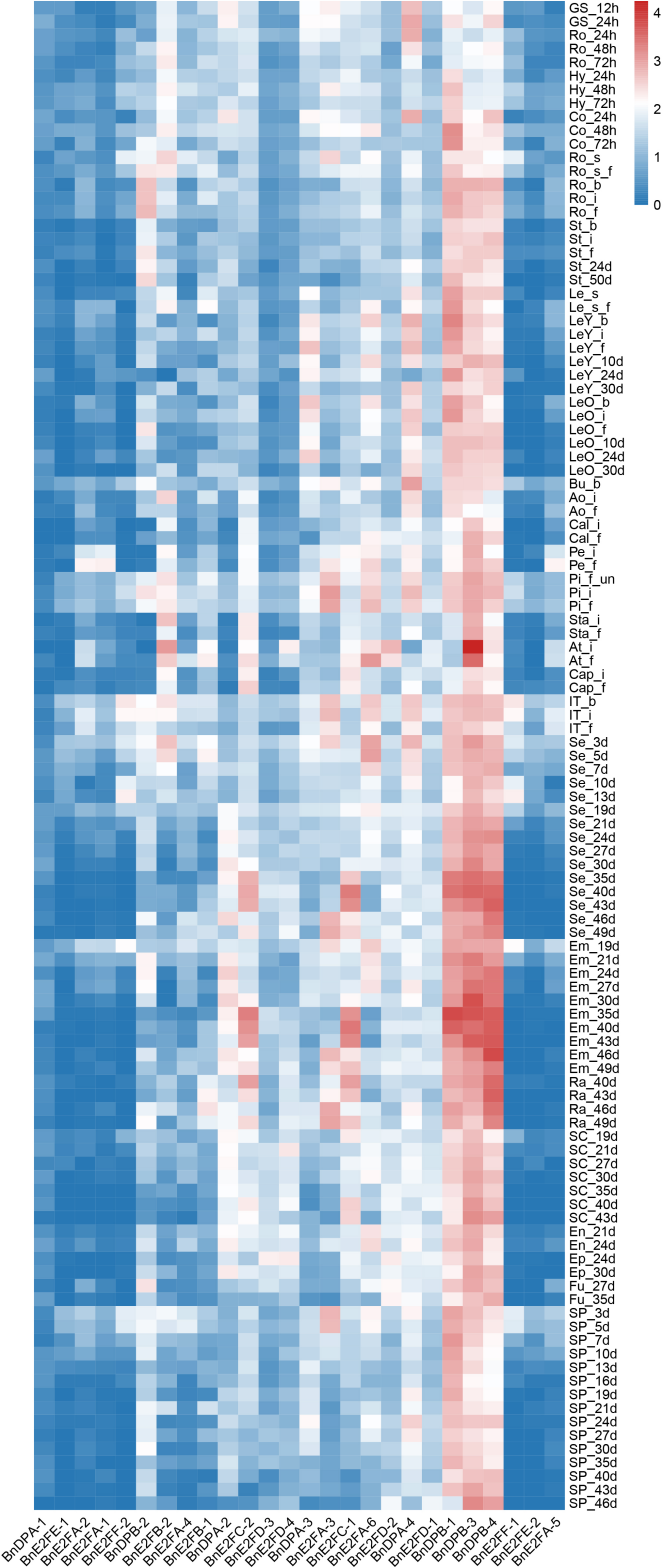
The tissue expression pattern of the *BnE2F/DP* genes.

In different tissue parts of plants, various genes play distinct roles and possess different functions. To conduct an in-depth exploration of the functions roles of *BnE2F/DP* genes, transcriptome data from *Brassica napus* were analyzed to characterize the expression profiles of *E2F/DP* family members across 24 vegetative and reproductive tissues, including cotyledons, hypocotyls, radicles and floral organs. Expression analyses were performed across 111 distinct tissues sampled at six developmental stages (germination, seedling, bud, initial flowering stage, full-bloom, and maturity). Based on the gene FPKM (Fragments Per Kilobase of exon model per Million mapped fragments) values obtained from the transcriptome data, expression profiles of 29 *BnE2F/DP* genes were generated and visualized (**Figure 6**). The heatmap analysis revealed significant variations in transcript abundance among *BnE2F/DP* family members, demonstrating spatiotemporal expression patterns. Notably, BnE2FC and BnDPB subfamilies genes displayed consistently high expressions across diverse tissues and organs, whereas BnE2FA, BnE2FE, and BnE2FF subfamily members showed generally low transcript levels. The remaining *BnE2F/DP* genes exhibited differential expression profiles across developmental stages and tissue types in *Brassica napus*. Collectively, *BnE2F/DP* gene family members displayed elevated transcript abundance in seeds and embryos, suggesting their involvement in reproductive development. Notably, 67% (2/3) of BnE2FA subfamily genes (BnE2FA-1, BnE2FA-2, BnE2FA-4, BnE2FA-5) showed negligible expression across most tissues, with only BnE2FA-3 and BnE2FA-6 displaying detectable transcripts. In contrast, BnE2FE and BnE2FF subfamilies each contained one silent member (BnE2FE-1 and BnE2FF-1, respectively) with consistently low transcript levels (FPKM < 0.5) throughout all developmental stages analyzed. In the BnDPB subfamily, 75% (3/4) of the genes (BnDPB-1, BnDPB-3, BnDPB-4) exhibited high FPKM values in almost all tissues and organs, indicating that the members of this sub-family might play a crucial role in the entire growth cycle of *Brassica napus*. This inference is consistent with the known functions of homologous genes in other plant species, which are often involved in fundamental cellular processes. In the BnE2FC subfamily, both genes (BnE2FC-1, BnE2FC-2) showed the highest FPKM values in seeds and embryos, suggesting their specific roles in seeds and embryos development.

In addition, BnE2FC-1, BnE2FC-2, BnE2FA-6, BnDPB-1, BnDPA-2, BnDPB-3, and BnDPB-4 in *Brassica napus* showed relatively high FPKM values in seeds from 10 to 49 days after pollination and in embryos from 19 to 49 days after pollination. Given that BnE2FC-1 and BnE2FC-2, BnDPB-1 and BnDPB-3, as well as BnDPB-4 belong to the same sub-family respectively, and considering that genes within the same sub-family often share conserved functional domains and participate in similar molecular pathways according to previous studies, it is suggested that they may have similar functions in *Brassica napus*.

### Validation of members of the *BnE2F/DP* gene family in *Brassica napus* by qRT-PCR

**Figure 7:**
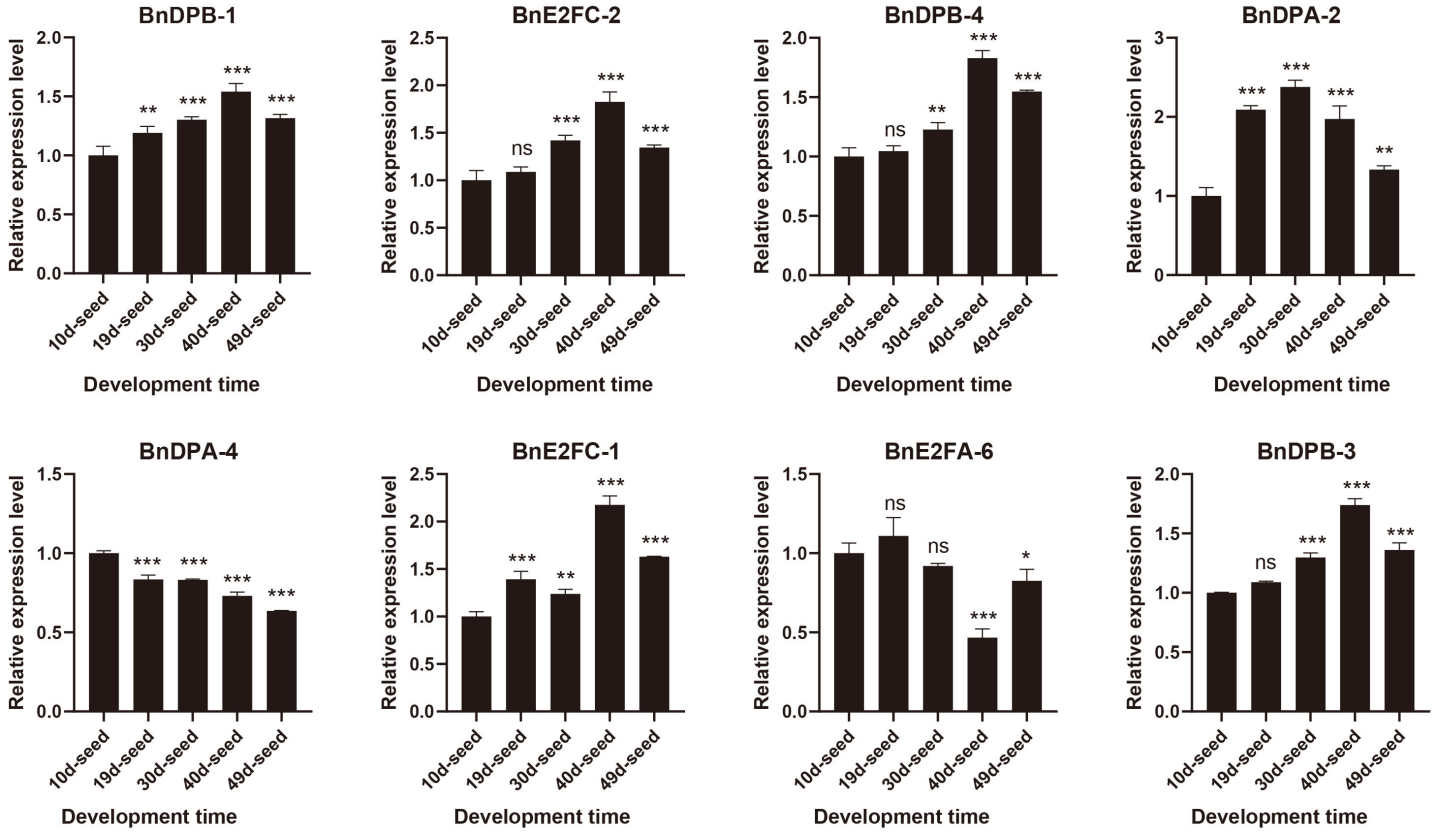
Real-time quantitative PCR was used to detect the expression of members of the *BnE2F/DP* gene family in seeds from 10 to 49 days after flowering. The qRT-PCR analysis was performed in triplicate, and the quantification was normalized using the 18S RNA level as a reference. The reported values are the mean, with error bars representing the standard error. Statistical significance was determined by Student's t-test, where *p < 0.05, *p < 0.01, *p < 0.001, and *p < 0.0001 indicate different levels of significance, and "ns" indicates no significant difference.

The results (**Figure 7**; **Supplementary Table 1**) confirmed high transcript levels of all eight selected genes across seed development, with *BnDPB-1, BnDPB-3*, and *BnDPB-4* showing peak expression at 40 days post-pollination. These results that *BnE2F/DP* genes play critical roles in seed development, consistent with transcriptome data and validated by qRT-PCR analysis.

### qRT-PCR validation of *BnE2F/DP* gene family members in *Brassica napus* under abiotic stresses

**Figure 8:**
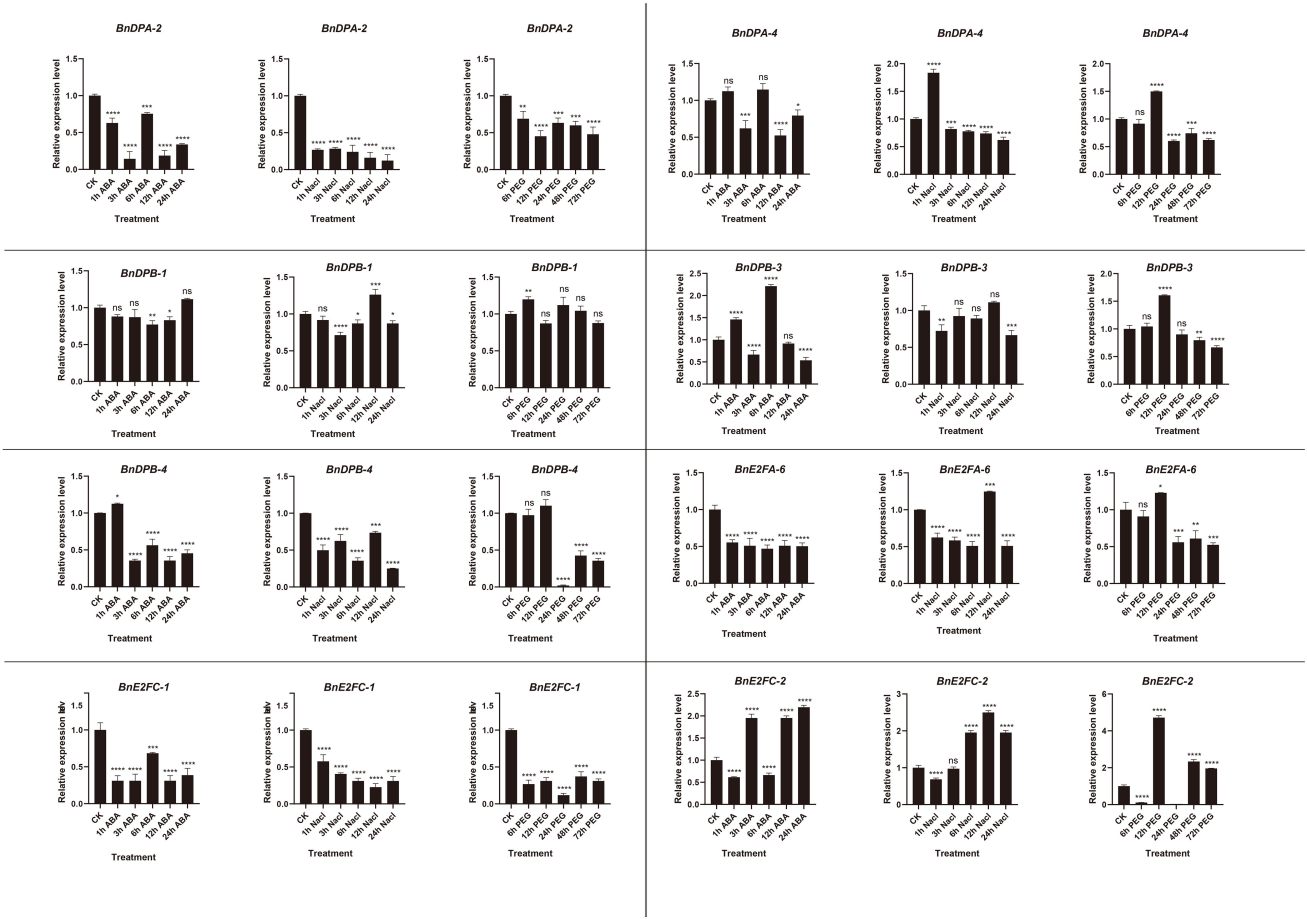
Real-time quantitative PCR (qRT-PCR) was used to detect the expression of *BnE2F/DP* gene family members at different time points under abiotic stresses induced by NaCl, ABA, and PEG.The reported values are the mean, with error bars representing the standard error. Statistical significance was determined by Student's t-test, *p < 0.05, *p < 0.01, *p < 0.001, and *p < 0.0001 indicate different levels of significance, and "ns" indicates no significant difference.

**Figure 9:**
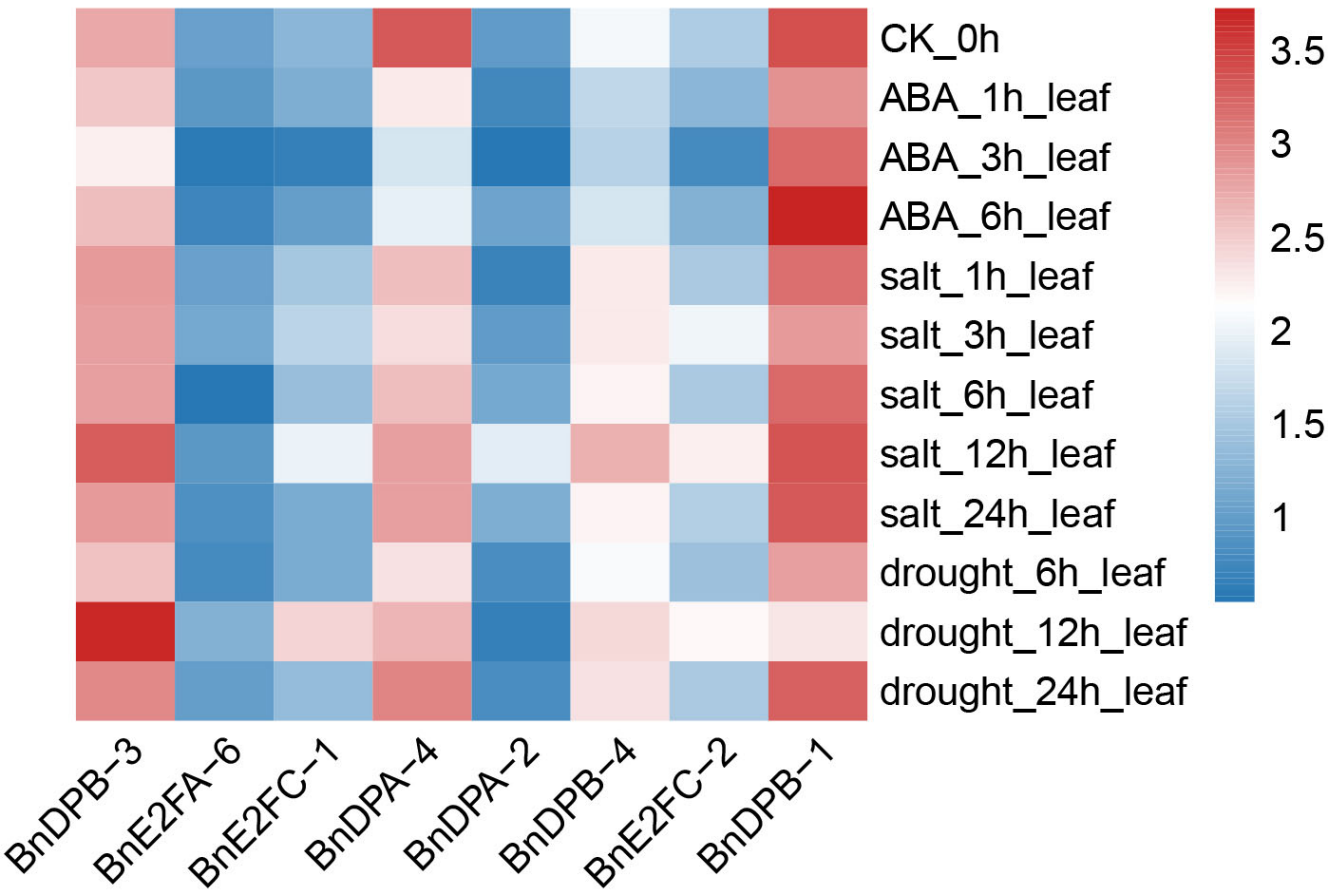
The expression pattern of the *BnE2F/DP* genes under abiotic stress.

Quantitative expression of eight genes under different treatments was detected with three replicates each. The results (**Figure 8**) showed that *BnE2F* genes were generally inhibited under ABA treatment, exhibiting a trend of first decreasing and then increasing. The gene expression levels peaked at 6 hours after treatment and then decreased, except for *BnDPB-1* and *BnE2FC-2*. Under salt stress, the expression of most genes was inhibited but increased after 12 hours, except for *BnDPA-2*, *BnDPA-4*, and *BnE2FC-1*. Under drought stress, gene expression peaked at 12 hours post-treatment and then rapidly declined, except for *BnDPA-2* and *BnDPB-1*. Notably, *BnDPA-2* and *BnE2FC-1* were consistently downregulated across all abiotic stress treatments and time points. The expression of *BnDPB-3* and *BnE2FC-2* was upregulated by more than two-fold under ABA treatment. Additionally, *BnDPA-4* and *BnE2FC-2* showed significant upregulation under salt stress, consistent with the expression patterns of *PheE2F/DP* genes in *Phyllostachys edulis* under salt stress (Li et al., 2021). The expression of *E2FC-2* was upregulated by more than two-fold at various time points under all three stresses, whereas *BnE2FC-1* was downregulated by nearly two-fold, consistent with the transcriptome data (**Figure 9**). These results suggest that the *BnE2FC* subfamily actively responds to diverse abiotic stresses, aligning with the predictions from cis-acting element analysis.

## Discussion

*E2F/DP* transcription factors are conserved regulators of plant cell cycle progression, photoperiodic responses, and seed/embryo development, while also mediating abiotic stresses (Mariconti et al., 2002; Lammens et al., 2009; Berckmans et al., 2011). In animal systems, they play critical roles in viral replication and host cell cycle manipulation during adenovirus infection. Investigating the functional roles of *E2F/DP* transcription factor genes is critical for deciphering plant development regulatory circutry. Recent high-throughput genomic approaches have enabled systematic characterization of *E2F/DP* gene families across diverse plant species, including *Phyllostachys heterocycla* (Pubescens), *Glycine max*, and *Solanum lycopersicum* (Bai et al., 2018). Nevertheless, the details of the *BnE2F/DP* gene family in *Brassica napus* remain poorly understood.

Genome-wide characterization of the *BnE2F/DP* gene family identified 29 members, representing a substantial expansion compared to other plant species: *Arabidopsis thaliana* (8 genes) (Xiaozhen et al., 2018), *Solanum lycopersicum* (8 genes) (Divya et al., 2024), *Triticum aestivum* (27 genes) (Yu et al., 2022), Zea mays (21 genes) (Lescot et al., 2002), *Medicago sativa* (Ma et al., 2014) (5 genes), *Phyllostachys edulis* (23 genes) (Li et al., 2021), and *Daucus carota* (10 genes) (Perrotta et al., 2021). The *Phyllostachys edulis* genome (2021 Mb) is slightly smaller than *Zea mays* (2300 Mb), significantly smaller than *Triticum aestivum* (17 Gb), and substantially larger than *Brachypodium distachyon* (300 Mb). The expanded Phe*E2F/DP*s gene family in *Phyllostachys edulis* (23 members) suggests that gene family size correlates with genome duplication events rather than the genome size. This pattern aligns with the differential gene expansion observed across plant lineages. Notably, the *Brassica napus* genes arose from an additional whole-genome triplication(WGT) event subsequent to its divergence from *Arabidopsis thaliana*, further illustrating the role of polyploidization in shaping gene family evolution. *Brassica napus* is an allopolyploid formed by the interspecific hybridization of *Brassica rapa* (AA genome) and *Brassica oleracea* (CC genome). Given the paleo hexaploid nature of both progenitor species (each having undergone a whole-genome triplication event subsequent to their divergence from *Arabidopsis thaliana*) theoretical predictions suggest that *B. napus* should contain 48 *E2F/DP* orthologs (8 *Arabidopsis* genes × 3 copies in each diploid progenitor × 2 sub genomes). However, our genome-wide analysis identified only 29 *E2F/DP* genes, indicating significant gene loss during the evolutionary history of this allopolyploid crop. Moreover, collinearity analysis shows that the genes have undergone purifying selection during evolution, suggesting that due to functional divergence has driven the retention of critical family members in *Brassica napus* for environmental adaptation, while non-essential duplicates were lost through fractionation. Chromosomal localization analysis revealed that the *BnE2F/DP* genes are unevenly distributed across 15 of 19 chromosomes in *Brassica napus*.The discrepancies in genes number among different sub-families might be attributed to gene amplification and loss events that have occurred during evolution.

Gene structure dictated functional diversity, with exons number variation potentially contributing to evolutionary divergence. During evolutionary, BnE2F/DP subfamilies such as BnE2FC and BnE2FA have retained a conserved exon number, suggesting functional constraint. In contrast, the BnDPA subfamily underwent functional divergence, accompanied by exon loss, which may have enabled the acquisition of novel biological roles distinct from other subfamilies. Conserved motifs analysis revealed that all 29 *BnE2F/DP* genes possess the E2F-TDP domain, with additional domains present in specific subfamilies. Motif 1 is universally conserved across all *BnE2F/DP* proteins, indicating its critical role in the evolutionary maintenance of the gene family in *Brassica napus*. The high degree of motif conservation among family members suggests functional constraint, with most subfamilies sharing common regulatory modules. The presence of the conserved TDP domain (PF00207) in all members confirms their classification within the E2F/DP transcription factor family, while additional lineage-specific domains reflect subfamily diversification. Motif 1 likely corresponds to the conserved TDP domain of the *BnE2F/DP* genes, consistent with previous reports that all Sl*E2F/DP* proteins in *Solanum lycopersicum* harbor this domain (Divya et al., 2024). Even after the evolutionary divergence of monocots and dicots within the *E2F/DP* gene family, these conserved motifs remain similar (Divya et al., 2024). *BnE2F/DP* genes within the same subfamily exhibit high sequence similarity. For example, BnE2FC and BnE2FA subfamilies share four identical motifs.

In the course of evolution, gene duplication events are a major source of new genetic traits. Segmental duplication increases the number of genes in specific gene families, thereby enhancing plants’ adaptability to various environmental stresses. Synteny analysis identified 35 duplication events among 29 *BnE2F/DP* genes in *Brassica napus*, with 28 pairs classified as segmental duplications. Moreover, the *BnE2F/DP* genes participated in intragenomic replication in the form of whole-genome duplication. Two duplication pairs in the tomato *SlE2F/DP* gene family indicate that segmental gene duplication triggered the expansion of the tomato *E2F/DP* gene family during evolution (Divya et al., 2024). In the wheat *E2F/DP* gene family, there are multiple segmental duplication events, and chromosomal segmental duplication plays a crucial role in the expansion and evolution of the *E2F* family (Yu et al., 2022). Twelve pairs of duplicated genes have been observed in the *E2F/DP* gene family of *Phyllostachys edulis* (moso bamboo) (Li et al., 2021). It has been reported that the functional differentiation between segmentally duplicated genes is more significant. The occurrence of multiple duplication events indicates that the *BnE2F/DP* gene family has been continuously expanding during the evolutionary process of the *Brassica napus* genome. This is consistent with the research results of *Zea mays* (Lescot et al., 2002) and *Phyllostachys edulis* (Li et al., 2021), indicating conserved *E2F/DP* gene family expansion patterns across plant lineages. Ka/Ks ratio analysis of the duplicated *BnE2F/DP* gene revealed values<1 for all pairs, indicating that the *BnE2F/DP* genes mainly evolved under purifying selection pressure, which is consistent with the evolutionary pattern of most gene families. Interspecific synteny analysis identified 9 orthologous genes pairs between *Arabidopsis thaliana* At*E2F/DP* and *Brassica napus BnE2F/DP*, suggesting shared ancestry and functional conservation.

It has been reported that members of the *E2F/DP* family in some plant species actively respond to abiotic stresses. Analysis of *Brassica napus BnE2F/DP* promoters revealed significant variation in the number and types of cis-regulatory elements across the 29 family members. However, all members contain 3 to 13 light-responsive elements, suggesting their involvement in light signaling pathways. This finding aligns with the previous reports that most PheE2FDPs in *Phyllostachys edulis* are regulated by the diurnal cycle (Li et al., 2021), implying that light plays a broad and fundamental role in modulating *BnE2F/DP* expression. Cis-regulatory elements analysis related that 28 *BnE2F/DP* promoters contain abiotic stress-responsive motifs, second only to light-responsive elements in abundance. This suggested that the *BnE2F/DP* family plays a broad role in *Brassica napus* stress responses, potentially mediating mechanisms of environmental sensing, signal transduction, and adaptive responses thereby helping the plants to adapt to external stresses and maintain normal physiological functions. Twenty-five *BnE2F/DP* genes contain cis-acting elements related to hormone responses, mainly involving abscisic acid, methyl jasmonate, gibberellin, salicylic acid, and auxin response elements. Notably, these genes exhibited significant enrichment in ABRE (ABA-responsive element) and MeJA-responsive motifs, with additional detection of abscisic acid, auxin, salicylic acid, and gibberellin-responsive elements in specific members. This suggests that their potential involvement in integrating multiple hormonal signaling pathways under environmental stresses. QRT-PCR analysis following abiotic stress treatments revealed that most *BnE2F/DP* genes exhibited high expression levels under both salt and drought stress, consistent with the expression patterns observed in the transcriptome data suggesting their potential roles in abiotic stress tolerance*BnDPA-4* and *BnE2FC-2* showed significant up-regulation under salt stress. Previous studies have found that *SlE2F/DP2*, *SlE2F/DP7*, and *SlE2F/DP8* members in tomato (*Solanum lycopersicum*) exhibit high expression under salt stress (Divya et al., 2024); *E2Fa*, *E2Fb*, *E2Fc*, and *E2FDPa* in alfalfa (*Medicago sativa*) show more than two-fold up-regulation in expression under salt stress, meanwhile after 6 hours of salt stress, the expression of *E2Fc* increases in leaves, while in roots, its expression first rises and then declines (Ma et al., 2014). In *Arabidopsis thaliana*, studies report changes in expression at 6 h and 24 h under salt stress, indicating that salt stress can trigger co-expression of *E2F* and *DP* genes (Ramirez-Parra et al., 2004; Desvoyes et al., 2006; Sozzani et al., 2006). The *TaDP2III-3* and *TaDEL2II-27* genes in wheat (*Triticum aestivum*) were upregulated under salt stress, while *TaE2F1I-19* and *TaDP3III-15* were up-regulated under drought stress (Yu et al., 2022). These results are consistent with the findings in *Phyllostachys edulis* (moso bamboo), where *PheE2F/DP* genes exhibited upregulation at various time points in response to both drought and salt stresses (Li et al., 2021).Notably, *BnE2FC-2* exhibited over two-fold up-regulation at different time points across all three stresses, while *BnE2FC-1* and *BnDPA-2* were repressed by nearly two-fold at various time points under the three stresses. These results suggest that the *BnE2FC* subfamily actively responds to diverse abiotic stresses, which is consistent with the predictions from the preceding cis-acting element analysis. The expression of *BnDPB-3* and *BnE2FC-2* was up-regulated by more than two-fold under ABA treatment. This observation aligns with the previous studies reporting significant enrichment in ABRE and MeJA response elements with *Phyllostachys edulis* Phe*E2F/DP* genes and tomatoes Sl*E2F/DP* genes (Li et al., 2021; Divya et al., 2024). Notably, plant genes expression is modulated not only by cis-regulatory elements but also by additional factors such as transcription factors and epigenetic modifications. Functional annotation of the BnDPB-4 promoter revealed an absence of development-related cis-regulatory elements but enrichment in hormone-responsive elements. This suggests that BnDPB-4 primarily mediates hormone signaling pathways in *Brassica napus* rather than participating in development process (Lescot et al., 2002).

Quantitative real-time PCR (qRT-PCR) validation exhibited high consistency with developmental transcriptome data. Throughout the developmental stages of *Brassica napus*, *BnE2FA* and *BnE2FE* subfamilies genes exhibited consistently low expression levels. This observation suggests limited or no significant roles for these genes during the growth and development. In contrast, *BnE2FC* and *BnDPB* subfamily members displayed constitutive high expressions across tissues (roots, leaves, flowers, and seeds) and developmental stages (**Figure 6**). This is consistent with the results that AtE2F in *Arabidopsis thaliana* (Xiaozhen et al., 2018) and DcE2F1 in *Daucus carota* (Perrotta et al., 2021) activate cell cycle genes in meristematic cells to promote cell mitotic proliferation. In tomato (*Solanum lycopersicum*), Sl*E2F/DP*3, Sl*E2F/DP*6, and Sl*E2F/DP*8 are mainly expressed in vegetative tissues (roots), while Sl*E2F/DP*1 and Sl*E2F/DP*7 displayed reproductive tissue-specific expression (e.g., flowers, immature fruits). The remaining genes showed constitutive expression across both vegetative and reproductive tissues. Sl*E2F/DP* genes play a regulatory role in the development, enlargement, and ripening processes of tomato fruits. This indicates that these genes may be involved in cell division and maturation processes (Li et al., 2021). A similar trend can be observed in the expression of *E2F/DP* genes in *Medicago sativa* (Ma et al., 2014). AtE2FC is generally regarded as a major repressor of E2F-regulated genes. Its overexpression reduces cell division and leads to an increase in endoreplication. It is hypothesized that BnE2FC may guide cells from division to differentiation in the late stage of seed maturation, thereby promoting the enlargement of seed cells and the accumulation of contents (Xiaozhen et al., 2018). In *Daucus carota*, DcE2F4, a homolog of AtE2FC, is reported to be well-expressed in young leaves, calli and other differentiated tissues, and is involved in regulating cell proliferation and the activities of mature cells (Perrotta et al., 2021). In summary, we speculate that the BnE2FC subfamily exhibits specific and high-level expression in seeds and embryos of rapeseed varieties, and shows a highly active response to abiotic stresses. Functional verification indicates that they play a key role in the development of seeds and embryos, and actively respond when plants are subjected to salt stress, ABA stress, and drought stress. This contrasts with previous findings in *Arabidopsis thaliana* (Xiaozhen et al., 2018), where overexpression of AtE2FA or AtE2FB significantly increased cotyledon size and doubled cells number. These observations suggest that during the evolution, homologous recombination events during gene duplication may have resulted in functional divergence of the *BnE2FC* subfamily in *Brassica napus*. These results suggest conserved roles for orthologous *BnE2F/DP* genes in regulating tissues and organ development across *Brassica napus* growth stages.

## Conclusion

In this study, 29 members of the *BnE2F/DP* gene family were identified in *Brassica napus* and classified into 8 subfamilies based on phylogenetic analysis. Functional annotation revealed abundant cis-regulatory elements within *BnE2F/DP* genes, which are involved in processes including growth and development, abiotic stress responses, hormone signaling, and light perception. Notably, all 29 gene members harbored light-responsive elements, suggesting their critical roles in mediating light signaling pathways in *Brassica napus*. Additionally, 28 genes contained abiotic stress-responsive cis-regulatory elements, indicating their significant involvement in mediating stress tolerance mechanisms under adverse environmental conditions.

Collinearity and duplication pattern analyses revealed that genome expansion of the *BnE2F/DP* gene family in *Brassica napus* occurred primarily occurred through whole-genome duplication events, with 35 collinear gene pairs identified. This finding reflects strong evolutionary conservation among family members. Ka/Ks ratio analysis further indicated that *BnE2F/DP* genes have undergone purifying selection during evolution. Transcriptomic analysis and qRT-PCR validation demonstrated that *BnE2F/DP* genes play pivotal regulatory functions across *Brassica napus* developmental stages, with particularly high expression during seed and embryo development. This highlights their active involvement in coordinating reproductive growth processes, including seed maturation and embryo patterning. Transcriptome prediction under stress and fluorescence quantitative PCR analysis revealed that members of the *BnE2F/DP* gene family exhibited upregulated or even highly expressed patterns after salt stress and drought stress treatments at different time points. *BnE2FC-2* showed more than two-fold high expression under different stresses and time points, indicating that the *BnE2FC* subfamily actively participates in stress responses, which is consistent with the previous cis-element prediction results. We speculate that *BnE2FC-2* is a candidate gene for salt and drought tolerance.

In summary, this study performed genome-wide identification and systematic bioinformatics analysis of the *BnE2F/DP* family in rapeseed (*Brassica napus*), with a focus on analyzing their expression patterns during growth and development stages and under abiotic stresses. The results provide insights for further functional studies of the *BnE2F/DP* gene family and identify potential candidate genes for genetic improvement of rapeseed in regulating growth and development as well as enhancing abiotic stress resistance. This study also offers new perspectives for a better understanding of the *BnE2F/DP* family in crops.

## Data Availability

The datasets presented in this study can be found in online repositories. The names of the repository/repositories and accession number(s) can be found in the article/**Supplementary Material**.
