# Supplementary material for: Identification and expression analysis of the BnE2F/DP gene family in Brassica napus
Source: Front Plant Sci. 2025 Aug 20;16:1641897. doi: 10.3389/fpls.2025.1641897 (PMC12405181; doi:10.3389/fpls.2025.1641897)
Supplement: Supplementary file 1 [file Table1.docx]

**Supplementary Table1 Summary of qRT-PCR gene expression of *BnE2F/DP* genes under different stress treatments**

| **Teat ment** | **1h** | | **3h** | | | **6h** | | **12h** | | **24h** | | **48h** | | **72h** | |  |
| --- | --- | --- | --- | --- | --- | --- | --- | --- | --- | --- | --- | --- | --- | --- | --- | --- |
|  | **up** | **down** | | **up** | **down** | **up** | **down** | **up** | **down** | **up** | **down** | **up** | **down** | **up** | **down** |  |
| **NaCl** | BnDPPA-4^***^ | BnDPA-2^****^  BnE2FC-1^***^  BnDPB-4^****^  BnE2FA-6^****^  BnE2FC-2^****^ | |  | BnDPA-2^,^BnDPA-4^,^BnDPB-1^,^  BnDPB-4^,^ BnE2FA-6,BnE2FC-1 | BnE2FC-2^****^ | BnDPA-2^****^ | BnE2FA-6^***^ | BnDPA-2^****^ | BnE2FC-2^****^ | BnDPA-2^****^ |  |  |  |  |  |
|  |  |  |  |  |  |  | BnDPA-4^***^ | BnE2FC-2^****^ | BnDPA-4^***^ |  | BnDPA-4^****^ |  |  |  |  |  |
|  |  |  |  |  |  |  | BnDPB-4^****^ |  | BnDPB-4^***^ |  | BnDPB-4^****^ |  |  |  |  |  |
|  |  |  |  |  |  |  | BnE2FA-6^****^ |  | BnE2FC-1^****^ |  | BnE2FA-6^****^ |  |  |  |  |  |
|  |  |  |  |  |  |  | BnE2FC-1 |  |  |  | BnE2FC-^1****^ |  |  |  |  |  |
| **PEG** |  |  | |  |  | BnE2FC-2^****^ | BnE2FC-1^****^ | BnE2FC-2^****^ | BnE2FC-1^****^ |  | BnDPA-2^***^ |  | BnDPA-2^***^ |  | BnDPA-2^****^ |  |
|  |  |  | |  |  |  |  | BnDPA-4^***^ | BnDPA-2^****^ |  | BnE2FC-1^****^ |  | BnE2FC-1^****^ |  | BnE2FC-^1****^ |  |
|  |  |  | |  |  |  |  | BnDPB-4^****^ |  |  | BnDPA-4^****^ |  | BnDPA-4^***^ |  | BnDPA-4^****^ |  |
|  |  |  | |  |  |  |  |  |  |  | BnDPB-4^****^ |  | BnDPB-4^****^ |  | BnDPB-4^****^ |  |
|  |  |  | |  |  |  |  |  |  |  | BnE2FA-6^***^ |  | BnE2FA-6^***^ |  | BnE2FA-6^***^ |  |
|  |  |  | |  |  |  |  |  |  |  | BnE2FC-2 |  | BnE2FC-2^****^ |  | BnE2FC-2^****^ |  |
| **ABA** | BnDPB-3^****^ | BnDPA-2^****^ | | BnE2FC-2^****^ | BnDPA-2^****^ | BnDPB-3^****^ | BnDPA-2^***^ | BnE2FC-2^****^ | BnDPA-2^****^ | BnE2FC-2^****^ | BnDPA-2^****^ |  |  |  |  |  |
|  |  | BnE2FC-1^****^ | |  | BnDPA-4^***^ |  | BnDPB-4^****^ |  | BnDPA-4^****^ |  | BnDPB-3^****^ |  |  |  |  |  |
|  |  | BnE2FA-6^****^ | |  | BnDPB-3^****^ |  | BnE2FA-6^****^ |  | BnDPB-4^****^ |  | BnDPB-4^****^ |  |  |  |  |  |
|  |  | BnE2FC-2^****^ | |  | BnDPB-4^****^ |  | BnE2FC-1^***^ |  | BnE2FA-6^***^ |  | BnE2FA-6^***^ |  |  |  |  |  |
|  |  |  | |  | BnE2FC-1^****^ |  | BnE2FC-2^****^ |  | BnE2FC-1^****^ |  | BnE2FC-1^****^ |  |  |  |  |  |
|  |  |  | |  | BnE2FA-6^****^ |  |  |  |  |  |  |  |  |  |  |  |
